# Thromboembolic and neurologic sequelae of discontinuation of an antihyperlipidemic drug during ongoing warfarin therapy

**DOI:** 10.1038/s41598-017-18318-6

**Published:** 2017-12-21

**Authors:** Charles E. Leonard, Colleen M. Brensinger, Warren B. Bilker, Stephen E. Kimmel, Heather J. Whitaker, Sean Hennessy

**Affiliations:** 1 Center for Clinical Epidemiology and Biostatistics, Department of Biostatistics, Epidemiology, and Informatics, Perelman School of Medicine at the University of Pennsylvania, Philadelphia, Pennsylvania USA; 2 Center for Pharmacoepidemiology Research and Training, Department of Biostatistics, Epidemiology, and Informatics, Perelman School of Medicine at the University of Pennsylvania, Philadelphia, Pennsylvania USA; 3 Center for Therapeutic Effectiveness Research, Department of Biostatistics, Epidemiology, and Informatics, Perelman School of Medicine at the University of Pennsylvania, Philadelphia, Pennsylvania USA; 4 Department of Psychiatry, Perelman School of Medicine at the University of Pennsylvania, Philadelphia, Pennsylvania USA; 5 Division of Cardiovascular Medicine, Department of Medicine, Perelman School of Medicine at the University of Pennsylvania, Philadelphia, Pennsylvania USA; 6 School of Mathematics and Statistics, The Open University, Milton Keynes, England; 7 Department of Systems Pharmacology and Translational Therapeutics, Perelman School of Medicine at the University of Pennsylvania, Philadelphia, Pennsylvania USA

## Abstract

Warfarin and antihyperlipidemics are commonly co-prescribed. Some antihyperlipidemics may inhibit warfarin deactivation via the hepatic cytochrome P450 system. Therefore, antihyperlipidemic discontinuation has been hypothesized to result in underanticoagulation, as warfarin metabolism is no longer inhibited. We quantified the risk of venous thromboembolism (VTE) and ischemic stroke (IS) due to statin and fibrate discontinuation in warfarin users, in which warfarin was initially dose-titrated during ongoing antihyperlipidemic therapy. Using 1999–2011 United States Medicaid claims among 69 million beneficiaries, we conducted a set of bidirectional self-controlled case series studies—one for each antihyperlipidemic. Outcomes were hospital admissions for VTE/IS. The risk segment was a maximum of 90 days immediately following antihyperlipidemic discontinuation, the exposure of interest. Time-varying confounders were included in conditional Poisson models. We identified 629 study eligible-persons with at least one outcome. Adjusted incidence rate ratios (IRRs) for all antihyperlipidemics studied were consistent with the null, and ranged from 0.21 (0.02, 2.82) for rosuvastatin to 2.16 (0.06, 75.0) for gemfibrozil. Despite using an underlying dataset of millions of persons, we had little precision in estimating IRRs for VTE/IS among warfarin-treated persons discontinuing individual antihyperlipidemics. Further research should investigate whether discontinuation of gemfibrozil in warfarin users results in serious underanticoagulation.

## Introduction

Drug-drug interactions are a serious public health problem. This problem is magnified in older adults, of whom >76% take two or more drugs^1^ and >50% take five or more drugs per month^2^. Given this degree of polypharmacy, it is not surprising that known drug-drug interactions are responsible for 13% of all adverse drug events^3^ and 4.8% of hospital admissions^4^ in older adults. Few studies have examined clinically-important population-based health effects of interactions between an object (the affected drug) and precipitant (the affecting drug)^5^. Even fewer (and possibly no) studies have examined clinical sequelae of an offset drug-drug interaction—triggered by discontinuation of a precipitant drug in the presence of ongoing object drug therapy (Appendix Figure 1)^6^. The paucity of data on the health effects of drug interactions leaves critical knowledge gaps for prescribers, pharmacists, nurses, patients, editors and users of drug interaction compendia, and persons who design, manage, and use clinical decision support systems.

Anticoagulants are consistently identified as among the most common causes of serious adverse drug events^7^. Underscoring this, the United States Department of Health and Human Services *National Action Plan for Adverse Drug Event Prevention* called for real world data on anticoagulant drug interactions^7^. Such interactions are of major concern since warfarin continues to be very commonly used (despite the rapid market uptake of direct oral anticoagulants)^8,9^, has a narrow therapeutic index^6^, may interact with almost every therapeutic class^10^, and is the leading cause of adverse drug event-related hospitalizations in older adults (the most common users of the drug^9^)^11^. Publically-available data from the United States Centers for Disease Control and Prevention^12^ indicate that nearly 50% of older adult warfarin users also take an antihyperlipidemic drug. Yet, intermittent use (e.g., suboptimal adherence) and long-term discontinuation (e.g., intolerance to adverse effects, perceived lack of efficacy, harms outweigh benefits) of antihyperlipidemic therapy is common^13,14^. For example, approximately 25–50% of statin users will discontinue their lipid-lowering drug within 6–12 months after initiation^15–17^. Antihyperlipidemic drug discontinuation may trigger an offset drug-drug interaction and thereby place persons at risk for sequelae of underanticoagulation.

The primary hypothesized mechanism underlying this putative offset drug-drug interaction involves de-inhibition of warfarin’s hepatic metabolism. When warfarin is initiated during ongoing use of an antihyperlipidemic drug, warfarin may be dose-titrated in the presence of an antihyperlipidemic that inhibits warfarin’s inactivation^18^ by cytochrome P450 2C9, 3A, and/or 1A2^19,20^. Therefore, less warfarin may be required to achieve a desired level of anticoagulation. Yet, upon discontinuing the antihyperlipidemic drug, metabolic inhibition is lost and the warfarin dose may be inadequate. Secondary mechanisms may include losses of the antihyperlipidemic’s plasma protein displacement of warfarin^21–23^ and pleotropic effects on platelets and the coagulation pathway^24–26^. As a result, such warfarin-treated patients may be underanticoagulated. To investigate this, we conducted a set of self-controlled case series studies to quantify and compare the rates of venous thromboembolism / ischemic stroke among concomitant users of warfarin upon discontinuation of individual antihyperlipidemic drugs.

## Results

In our dataset of over 69 million beneficiaries, we identified 629 subjects who: a) concomitantly used warfarin and an antihyperlipidemic of interest; b) experienced at least one venous thromboembolism / ischemic stroke outcome during observation time; and c) met all other inclusion criteria. Subjects were predominantly female (65.0%) and non-Hispanic Caucasian (56.9%), with a median age of 69.1 years. Subjects contributed 93,764 person-days of observation, 84,208 (89.8%) of which were in a non-hospital setting. The median and mean observation period length was 3.3 and 4.9 months, respectively. The risk, non-risk, and indeterminate risk segments accounted for 12.5%, 84.3%, and 3.2% of person-days, respectively. Subject characteristics stratified by antihyperlipidemic of interest are presented in Table 1. Note that there were ten or fewer persons constituting the cerivastatin, fluvastatin, and pitavastatin cohorts; therefore, self-controlled case series conditional Poisson regression models were not run for these agents.

**Table 1:** Characteristics of warfarin users under study, by antihyperlipidemic cohort.

		Antihyperlipidemic of interest									
		atorva	ceriva*	feno	fluva*	gem	lova	pitava*	prava	rosuva	simva
Persons		219	**	24	**	11	36	**	68	22	235
Persons-days of observation period, median (Q1-Q3) per individual		112.0 (45.0–197.0)	79.5 (31.5–128.0)	98.0 (57.5–217.0)	66.0 (43.0–110.0)	43.0 (29.0–126.0)	74.5 (40.5–189.0)	17.0 (17.0–17.0)	102.0 (53.0–203.5)	109.0 (57.0–171.0)	100.0 (46.0–190.0)
Person-days of observation period, total		34,789	319	3,772	1,156	811	4,726	17	10,610	2,585	34,979
% person-days of observation period in risk segment		13.5	23.2	13.2	23.8	28.0	12.5	0.0	12.6	11.7	10.6
Outcomes during observation period		238	**	27	11	11	38	**	73	23	248
VTE		170	**	**	**	**	26	**	52	**	162
IS		68	0	**	**	**	12	0	21	**	86
**Demographics**	**Group**	**% of persons (unless otherwise noted)**									
Age in years at start of observation period	Median (Q1-Q3)	68.9 (54.3–78.1)	80.5 (72.0–86.5)	61.3 (48.4–69.9)	66.5 (51.8–84.0)	57.3 (46.1–63.4)	72.2 (57.1–80.2)	62.5 (62.5–62.5)	71.4 (57.2–78.7)	63.8 (55.3–74.4)	71.1 (59.4–80.0)
Sex	Female	69.9	**	66.7	**	**	61.1	**	58.8	77.3	62.6
Race	White	60.3	**	79.2	**	**	55.6	0.0	60.3	**	51.9
	Black	16.9	**	**	0.0	**	**	**	**	**	23.8
	Hispanic/Latino	8.7	0.0	0.0	**	**	**	0.0	**	**	11.5
	Other/Unknown	14.2	**	**	**	**	**	**	**	**	12.8
State of residence	California	39.3	**	**	**	**	41.7	0.0	54.4	**	29.8
	Florida	14.2	**	**	**	**	**	**	17.6	**	17.9
	New York	24.2	0.0	**	**	**	**	0.0	**	**	26.0
	Ohio	13.2	**	**	**	**	**	**	**	**	12.3
	Pennsylvania	9.1	0.0	**	**	**	**	0.0	**	**	14.0
Calendar year at start of observation period^†^	1999	**	**	**	**	0.0	**	0.0	**	0.0	**
	2000	**	**	0.0	**	**	**	0.0	**	0.0	**
	2001	10.0	**	**	**	**	**	0.0	**	0.0	5.5
	2002	9.6	0.0	0.0	0.0	**	**	0.0	**	0.0	4.7
	2003	7.3	0.0	**	**	0.0	**	0.0	**	0.0	4.7
	2004	8.2	0.0	0.0	**	**	**	0.0	**	0.0	**
	2005	10.5	0.0	**	0.0	**	**	0.0	**	**	**
	2006	12.3	0.0	**	0.0	0.0	**	0.0	**	**	4.7
	2007	9.1	0.0	**	**	0.0	**	0.0	**	0.0	11.5
	2008	10.5	0.0	**	0.0	**	**	0.0	**	**	13.6
	2009	8.7	0.0	**	0.0	**	**	0.0	**	**	14.5
	2010	5.5	0.0	**	0.0	**	**	0.0	**	**	17.4
	2011	**	0.0	**	0.0	0.0	**	**	**	**	14.9
Medicare enrolled at start of observation period	Yes	79.0	**	75.0	**	**	88.9	**	88.2	81.8	77.4
Nursing home resident at start of observation period		8.7	**	**	**	**	**	**	**	**	17.4
**Pre-defined time-varying covariates**	**Group**	**% of person-days (unless otherwise noted)**									
Major non-chronic risk factor for outcome											
VTE in prior 90 days	Yes	30.3	68.0	54.9	17.9	69.2	32.2	**	30.9	38.4	28.9
IS in prior 90 days		9.0	0.0	3.2	12.5	5.5	7.5	0.0	8.5	20.0	12.4
Hospital discharge on current day or in prior 90 days		31.6	49.2	41.6	35.7	70.4	36.1	**	30.2	41.5	34.2
Major non-chronic disease that may affect coagulation											
Acute infection on current day or in prior 14 days	Yes	15.3	16.9	10.5	15.6	14.1	10.5	**	16.7	16.1	15.3
Drug that may affect coagulation^†^											
anticoagulant, oral non-warfarin	Yes	0.0	0.0	0.0	0.0	0.0	0.0	0.0	0.0	0.0	0.0
anticoagulant, injectable/subcutaneous		0.9	0.0	0.0	0.0	0.0	0.0	0.0	0.8	1.2	0.5
antiplatelet, oral		0.0	0.0	0.0	0.0	0.0	0.0	0.0	0.0	0.0	0.0
aspirin		0.0	0.0	0.0	0.0	0.0	0.0	0.0	0.0	0.0	0.0
Drug that may interact with warfarin											
interacting drug, oral, per Truven^‡^	Yes	29.3	42.9	30.4	10.4	26.3	38.6	0.0	30.7	12.4	17.3
CYP2C9 inhibitor^‡^		11.0	42.6	15.6	0.0	7.5	10.3	0.0	8.4	2.3	5.0
CYP2C9 inducer^†^		5.8	0.0	0.0	0.0	0.0	0.6	0.0	6.1	0.0	1.6
Drug that may increase risk of VTE alone^†^	Yes	26.7	0.0	31.0	11.5	55.7	40.3	0.0	19.5	19.1	21.9
Drug that may increase risk of IS alone^‡^	Yes	24.8	42.6	30.0	3.1	9.0	25.0	0.0	19.5	13.2	23.8
Drug that may increase risk of VTE and IS^†^	Yes	15.9	46.1	17.7	12.8	24.7	26.2	0.0	19.1	18.8	10.2
Therapeutic drug monitoring for warfarin	Yes	32.5	41.1	31.8	27.3	29.1	28.0	0.0	31.0	31.8	31.1
Average daily warfarin dose, in milligrams	Median (Q1-Q3)	4.8 (2.5–5.0)	4.0 (4.0–4.0)	3.0 (2.0–5.0)	4.0 (2.5–5.0)	5.0 (3.7–6.0)	5.0 (2.5–5.0)	7.5 (7.5–7.5)	2.5 (1.5–5.0)	3.0 (2.0–5.0)	4.0 (2.0–5.0)

Atorva = atorvastatin; CYP = cytochrome P450; feno = fenofibrate; gem = gemfibrozil; IS = ischemic stroke; lova = lovastatin; prava = pravastatin; Q = quartile; rosuva = rosuvastatin; simva = simvastatin; VTE = venous thromboembolism.

*Excluded from further study because few persons in cohort.

**Value suppressed to ensure subject anonymity, consistent with Centers for Medicare and Medicaid Services privacy rule for small cells; cell < 11 or would permit back-calculation of a cell < 11.

^†^If dispensed on current day or in prior 30 days.

^‡^If dispensed on current day or in prior 30 days for chronically-administered drugs (14 days for acutely-administered drugs).

**Figure 1:**
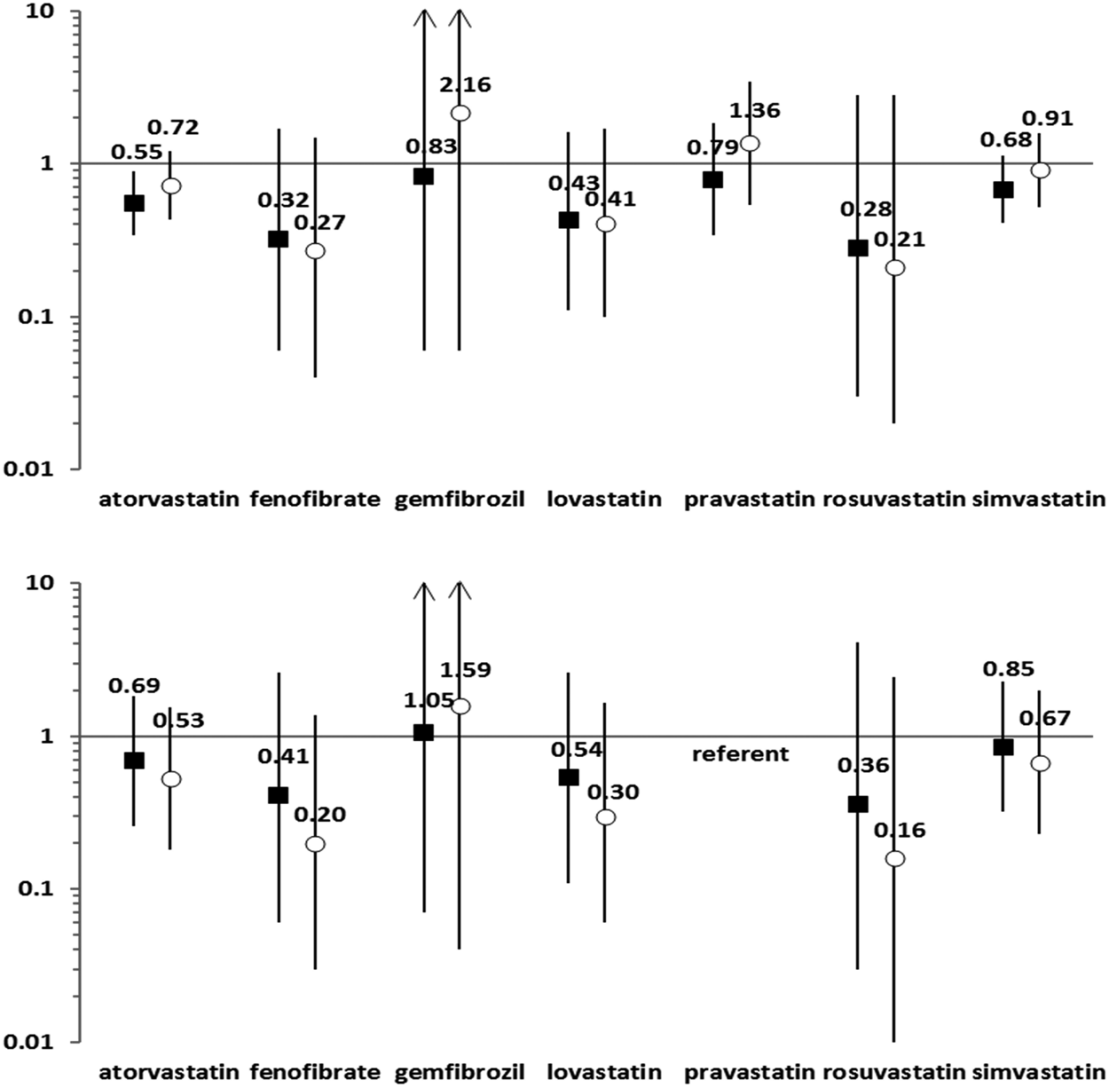
Risk of venous thromboembolism/ischemic stroke within 90 days of discontinuing an antihyperlipidemic of interest in the presence of ongoing warfarin therapy. Panel A (top): Crude and adjusted incidence rate ratios. Panel B (bottom): Ratio of crude and adjusted incidence rate ratios for antihyperlipidemic of interest vs. pravastatin. Figure 1 presents primary findings of study. Black squares represent crude incidence rate ratios. White circles represent confounder-adjusted incidence rate ratios. Values for incidence rate ratios and 95% confidence intervals are presented in Appendix Table 1.

For the primary analysis, the crude incidence rate ratios for venous thromboembolism/ischemic stroke within 90 days of antihyperlipidemic discontinuation ranged from 0.28 (0.03–2.80) for rosuvastatin to 0.83 (0.06–11.23) for gemfibrozil. Confounder-adjusted incidence rate ratios ranged from 0.21 (0.02–2.82) for rosuvastatin to 2.16 (0.06–75.00) for gemfibrozil. Crude and adjusted incidence rate ratios for pravastatin, the prespecified referent, were 0.79 (0.34–1.83) and 1.36 (0.54–3.45), respectively. See Fig. 1 and Appendix Table 1. Findings from secondary analyses are presented in Table 2 and Appendix Table 2.

**Table 2:** Findings from prespecified and post hoc secondary analyses.

Analysis*		Ratio of adjusted IRRs (95% CIs) for antihyperlipidemic of interest vs. referent						
		fibrates		statins				
		feno	gem	rosuva	atorva	prava	lova	simva
***Further elucidating the association between antihyperlipidemic discontinuation and outcome***								
Stratify risk segment	Days 1–30	0.25 (0.03–1.76)	5.11 (0.10–265.8)	0.21 (0.02–2.64)	0.44 (0.15–1.30)	referent	0.29 (0.06–1.47)	0.42 (0.14–1.32)
	Days 31–60	ND	ND	ND	ND	referent	ND	ND
	Days 61–90	ND	ND	ND	0.41 (0.03–5.63)	referent	ND	0.21 (0.01–5.33)
Deconstruct composite outcome	VTE	0.15 (0.01–1.77)	1.45 (0.05–41.1)	0.35 (0.02–7.56)	0.43 (0.12–1.54)	referent	0.63 (0.11–3.73)	0.65 (0.18–2.40)
	IS	0.45 (0.02–13.1)	ND	ND	0.88 (0.11–6.70)	referent	ND	0.47 (0.06–3.55)
Lump antihyperlipidemics of interest by likelihood of CYP2C9 inhibition**		0.71 (0.37–1.35)				referent		
Lump antihyperlipidemics of interest by likelihood of interacting with warfarin, per Truven Micromedex and Facts & Comparisons DDI module ratings**		0.86 (0.45–1.64)			referent		†	
***Assessing SCCS underlying assumptions***, ***minimizing the role of bias and/or confounding***								
Increase maximum length of risk segment from 90 to 120 days^††^		0.19 (0.03–1.32)	1.09 (0.02–65.0)	0.21 (0.02–2.04)	0.46 (0.16–1.29)	referent	0.39 (0.08–1.81)	0.53 (0.18–1.56)
Exclude segments occurring before the first risk segment (i.e., conduct left-censored unidirectional SCCS)		ND	ND	ND	0.71 (0.06–8.36)	referent	ND	0.36 (0.03–4.20)
Exclude segments occurring after the first risk and indeterminate risk segments (i.e., conduct right-censored unidirectional SCCS)		0.27 (0.03–2.49)	2.54 (0.06–104.6)	0.51 (0.03–7.94)	0.82 (0.26–2.59)	referent	0.85 (0.12–5.78)	1.01 (0.31–3.27)
Reclassify second or later risk and indeterminate risk segments as non-risk segments		0.22 (0.03–1.59)	1.51 (0.04–59.3)	0.29 (0.02–4.41)	0.54 (0.18–1.58)	referent	0.43 (0.08–2.14)	0.66 (0.22–1.98)
Include average daily dose of warfarin as covariate in outcome model		0.24 (0.03–2.09)	8.55 (0.28–259.0)	0.35 (0.02–5.79)	0.90 (0.27–3.03)	referent	0.37 (0.06–2.29)	0.97 (0.28–3.32)
Exclude subjects with >1 outcome during the observation period		0.37 (0.05–2.77)	2.42 (0.06–98.7)	0.34 (0.02–5.27)	0.86 (0.26–2.89)	referent	0.74 (0.12–4.42)	1.19 (0.35–4.02)
Exclude subjects that die during the observation period		0.21 (0.03–1.52)	1.19 (0.04–36.7)	0.15 (0.01–2.30)	0.43 (0.14–1.27)	referent	0.34 (0.06–1.86)	0.62 (0.20–1.90)

Atorva = atorvastatin; CI = confidence interval; CYP = cytochrome P450; DDI = drug-drug interaction; feno = fenofibrate; gem = gemfibrozil; IRR = incidence rate ratio; IS = ischemic stroke; lova = lovastatin; ND = not detectable/model produced unstable estimates; prava = pravastatin; rosuva = rosuvastatin; SCCS = self-controlled case series; simva = simvastatin; VTE = venous thromboembolism.

*Examining VTE/IS as composite outcome, unless otherwise noted.

**Post hoc analysis.

^†^IRR for combined fenofibrate/gemfibrozil/rosuvastatin/lovastatin/simvastatin vs. atorvastatin/pravastatin listed in merged fenofibrate-gemfibrozil-rosuvastatin cell.

^††^Thereby increases maximum length of indeterminate risk period from 90 to 120 days.

## Discussion

We examined rates of venous thromboembolism/ischemic stroke among users of warfarin upon discontinuation of concomitantly-prescribed antihyperlipidemic drugs. Using rigorous pharmacoepidemiologic methods and a dataset of healthcare claims from tens of millions of persons, we did not identify any statistically significant confounder-adjusted associations between antihyperlipidemic discontinuation and our composite outcome. Our findings’ limited precision prohibits us from drawing definitive conclusions; this is a notable limitation. However, it is worth noting that adjusted incidence rate ratios vs. pravastatin were less than one for fenofibrate and nearly always less than one for each statin under study. Results when combining antihyperlipidemics of interest by likelihood of cytochrome P450 2C9 inhibition using two different groupings resulted in substantial increases in precision and still failed to demonstrate a statistically significant association. Because the adjusted incidence rate ratio for gemfibrozil vs. pravastatin was 1.59, one could hypothesize that discontinuing gemfibrozil might increase one’s risk of venous thromboembolism/ischemic stroke. This finding aligns with our prior demonstration that warfarin plus gemfibrozil (vs. pravastatin) results in a 50% increased risk of gastrointestinal bleeding and intracranial hemorrhage^27^, a result of overanticoagulation. Therefore, if warfarin is dose-titrated in the presence of gemfibrozil exposure, a lower dose of warfarin may be required to reach the desired level of anticoagulation. If gemfibrozil therapy is subsequently discontinued, this may place the patient at risk for sequelae of underanticoagulation from suboptimal warfarin dosing. This may emphasize the importance of calls to monitor the level of anticoagulation upon initiating *and* discontinuing drugs that may inhibit warfarin’s metabolism^6,28,29^, including certain antihyperlipidemics^6^.

To our knowledge, no prior population-based comparative safety study has investigated clinical outcomes associated with the discontinuation of individual statins and/or fibrates in users of a coumarin derivative. With respect to a surrogate endpoint, Zhelyazkova-Savova *et al*. examined changes in laboratory measures in a cross-sectional study elucidating potential statin drug interactions (Appendix Table 3)^30^. Among 69 Bulgarian inpatients concomitantly-exposed to acenocoumarol and a statin, one (1.4%) individual experienced a 22.6% reduction in their international normalized ratio (3.1 to 2.4) upon discontinuation of atorvastatin^30^. In a cross-sectional study examining a potential drug interaction between warfarin and amiodarone (a non-statin, non-fibrate precipitant drug that inhibits the metabolism of warfarin via cytochrome P450, similar to some statins and fibrates), McDonald *et al*. quantified that amiodarone discontinuation necessitated a 24.8% mean increase in warfarin dose to maintain the international normalized ratio of 27 outpatients between 2.0 and 3.0 (Appendix Table 3)^31^. It is possible that amiodarone discontinuation during warfarin use may be more clinically relevant than the warfarin-antihyperlipidemic offset drug-drug interaction examined herein, particularly with respect to statin discontinuation. This warrants further investigation.

Our study has notable strengths. It is the first population-based comparative safety study to examine clinical sequelae of an offset drug interaction among warfarin users. We utilized a self-controlled study design, prespecified a reference exposure, and controlled for time-varying covariates to minimize confounding. We conducted numerous secondary analyses to further elucidate the association between exposure and outcome and to test the robustness of findings to assumptions of the self-controlled design. Finally, components of our outcome definition had high positive predictive values and moderate-to-high sensitivities.

Our study also has limitations. First, despite using a dataset of over 69 million individuals, we had limited statistical power. Second, we lacked access to biosamples and therefore could not examine the impact of genetic cytochrome P450 polymorphisms. Third, we lacked data on adherence to dispensed warfarin and antihyperlipidemic prescriptions. Fourth, we lacked access to results of laboratory orders (e.g. international normalized ratio values); such findings are not included in Centers for Medicare and Medicaid Services data. Fifth, administrative databases may poorly capture some lifestyle behaviors and nonprescription therapies that affect venous thromboembolism and/or ischemic stroke risk; yet, such factors seem unlikely to differ substantially by antihyperlipidemic exposure group. Finally, our results may not be generalizable beyond a United States Medicaid population. Nevertheless, this population was specifically chosen because of its inherent vulnerability and inclusion of large numbers of women and minorities—groups typically understudied. Biologic associations identified in Medicaid populations are often replicated in commercially insured populations and vice versa^32^.

Drug interactions with warfarin are a major public health concern. Nearly all existing population-based studies of warfarin interactions and clinical outcomes have examined risk periods defined by *commencement* of concomitant use of warfarin and a precipitant drug. In contrast, we examined putative offset drug-drug interactions—defined by *discontinuation* of the precipitant drug—hypothesizing that de-inhibition of warfarin’s hepatic metabolism would lead to serious sequelae of underanticoagulation. We did not identify a clear relationship between discontinuing antihyperlipidemics and clinical events. The potential safety signal that concomitant users of warfarin and gemfibrozil may be at increased risk for venous thromboembolism/ischemic stroke upon discontinuation of gemfibrozil requires further study. The mechanism underlying this possible offset interaction also needs further elucidation, but is unlikely to solely involve a pharmacokinetic interaction mediated by cytochrome P450 inhibition or displacement of binding from plasma proteins.

## Methods

### Overview and study population

We conducted bidirectional self-controlled case series studies of adult users of warfarin experiencing the composite outcome of venous thromboembolism/ischemic stroke. Although the phrase “case series” within self-controlled case series may seem to imply the absence of a comparator, the design is actually a rigorous, reproducible, controlled epidemiologic method^33^; it is the cohort analogue of the better known case-crossover design^34^. In a self-controlled case series study, individuals serve as their own referent, therefore eliminating confounding by time-invariant factors^35^. This is a major advantage over traditional cohort and case-control approaches, yet is accompanied by the following key assumptions: the occurrence of an outcome should not appreciably affect subsequent exposures; outcome rates are constant within intervals; and outcomes must be independently recurrent or rare^35^.

The study’s underlying cohorts, one for each of ten antihyperlipidemics of interest, consisted of episodes of new warfarin use initiated during ongoing therapy for that antihyperlipidemic. Study data included demographic, enrollment, and healthcare claims from the United States Medicaid programs of California, Florida, New York, Ohio, and Pennsylvania from 1999–2011^32^. These states comprise ~38% of the national enrollment^36^, with the 13-year dataset recording the experience of more than 69 million cumulative enrollees and nearly 222 million person-years of observation. Because a substantive proportion of Medicaid beneficiaries are co-enrolled in Medicare^37–39^, we included Medicare claims to ascertain a more complete picture of their healthcare^40,41^. We linked these datasets to the Social Security Administration Death Master File to supplement death dates included in Medicaid and Medicare enrollment files.

### Study cohorts

Separate cohorts were constructed for each antihyperlipidemic of interest, serving as bases for each self-controlled case series study. For persons 18–100 years of age, we utilized National Drug Codes and days’ supply values on prescription claims to build episodes of warfarin exposure. We allowed a 7-day grace period between contiguous warfarin prescriptions (and at the end of the terminal warfarin prescription) to account for imperfect adherence. This approach was repeated for each antihyperlipidemic of interest, thereby allowing us to identify persons concomitantly exposed to warfarin and an antihyperlipidemic. We then identified each person’s first concomitant use episode during which the antihyperlipidemic drug was initiated at least 30 days prior to warfarin; this ensured that warfarin was initially dose titrated while hepatic cytochrome P450 isozymes were already inhibited by the antihyperlipidemic (if applicable). Further, as the self-controlled case series design is a “case-only” approach, each person under study was required to experience an outcome during their observation period (defined below). If fewer than 10 persons constituted a given cohort, the antihyperlipidemic was excluded from further study.

### Observation and pre-observation baseline periods

For each cohort member meeting inclusion criteria, their observation period included all person-days of the warfarin episode that defined concomitancy. The observation period began upon warfarin initiation and was censored upon the earliest of: a) the end of the warfarin episode initially defining concomitancy (defined by exhausting days’ supply [plus terminal grace period] or switching to a different oral anticoagulant); b) a dispensing for an antihyperlipidemic other than that initially defining concomitancy; c) a >7-day gap in Medicaid enrollment; d) the end of the study dataset; and e) death. Note that occurrence of an outcome did not censor observation time. This helped uphold the key self-controlled case series assumption of no event-dependent censoring^42^ and thereby avoided introducing bias of an unpredictable direction^43^.

A baseline period was defined as the 180 days immediately before the observation period. It was required to be devoid of: a) a >7-day gap in Medicaid enrollment; b) a procedure code indicative of hepatic cytochrome P450 2C9 or vitamin K epoxide reductase complex genotyping—suggestive that the prescriber used genetics to guide warfarin dose titration; and c) a prescription claim for warfarin or any other oral anticoagulant—otherwise, a prescriber may not dose-titrate but rather reinstitute an old, tolerated dose of warfarin.

### Categorizing observation period follow-up time

**Figure 2:**
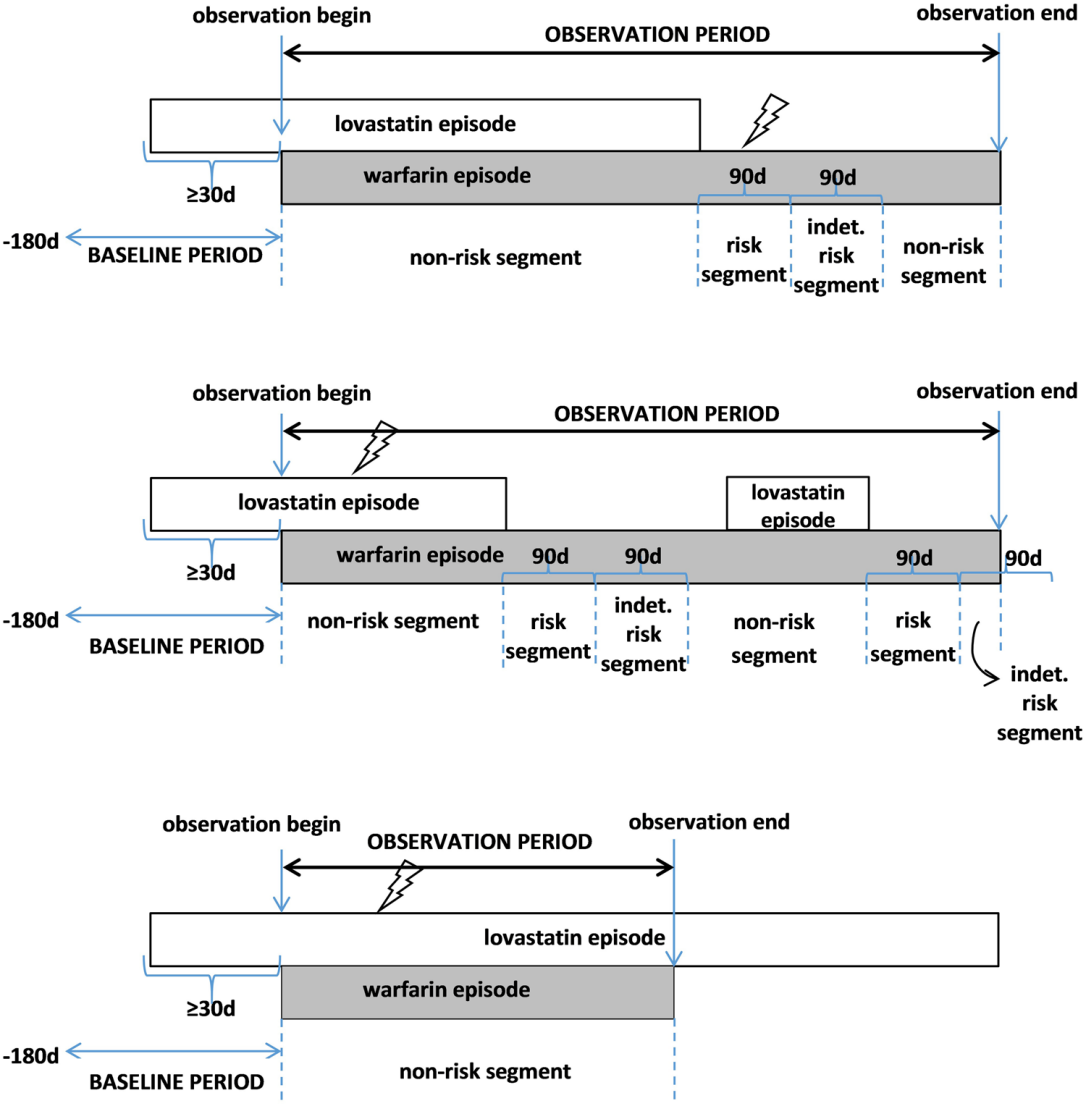
Examples of concomitant use episodes of warfarin and lovastatin eligible for inclusion. Panel A (top two): Persons with risk, indeterminate risk, and non-risk segments. Panel B (bottom): Person with non-risk segment only. Figure 2 presents potential methods of cohort entry, using concomitant use of warfarin and lovastatin as an example. Lightning bolts represent hypothetical outcomes.

Person-days within each observation period were assigned to mutually-exclusive risk, non-risk, and indeterminate risk segments—with risk segments commensurate with a biologically plausible time frame during which antihyperlipidemic discontinuation in the presence of warfarin may be expected to increase risk of the outcome^44,45^. The risk segment consisted of a maximum of 90 person-days immediately following the end of the antihyperlipidemic episode initially defining concomitancy. The indeterminate risk segment consisted of a maximum of 90 person-days immediately following the risk segment. These segments could be <90 days in length if censored (described above) or if the antihyperlipidemic initially defining concomitancy was re-initiated. The non-risk segment consisted of all other person-days of observation time not assigned to a risk or indeterminate risk segment. Therefore, non-risk segments could occur both before and after the risk segment, consistent with a bidirectional self-controlled case series design; this standard approach helped to minimize exposure trend bias^46^. Of note, each observation period was not required to have person-days in all segments, yet only observations with both risk and non-risk segments contributed to the estimation of the incidence rate ratio for the association of interest. See Fig. 2 for a graphical representation of concomitant use episodes potentially eligible for inclusion.

### Exposure of interest and covariates

The exposure of interest was the antihyperlipidemic drug initially defining concomitancy that was subsequently discontinued in the presence of warfarin. Agents included atorvastatin, cerivastatin, fenofibrate, fluvastatin, gemfibrozil, lovastatin, pitavastatin, pravastatin, rosuvastatin, and simvastatin. As dictated by the methodologic approach, each of the exposures of interest was examined in a separate self-controlled case series study. Pravastatin served as a prespecified control precipitant^5^ referent because it is a negligible inhibitor of hepatic cytochrome P450 isozymes^47^ involved in the metabolism of warfarin^18^ and thus not expected to interact pharmacokinetically. Consequently, the discontinuation of pravastatin during ongoing warfarin therapy (dose-titrated while pravastatin was on board) would not be expected to lead to underanticoagulation.

The self-controlled case series design implicitly controls for time-invariant covariates^48^. We therefore considered only time-varying covariates as potential confounders. We included, in each regression model, covariates from the following broad categories: a) major non-chronic risk factors for venous thromboembolism or ischemic stroke; b) drugs that may increase the risk of venous thromboembolism, ischemic stroke, or both^49^; c) major non-chronic diseases that may affect coagulation; d) drugs that may affect coagulation; e) drugs that may interact with warfarin^50,51^; and f) therapeutic drug monitoring for warfarin (i.e., an order for an international normalized ratio). We added a time-varying covariate for average daily warfarin dose in a secondary analysis; this was relegated to a secondary analysis since warfarin dose is difficult to ascertain from prescription dispensings. See Appendix Table 4 for additional detail.

### Outcome of interest

The composite outcome was hospitalization for venous thromboembolism or ischemic stroke—serious sequelae of underanticoagulation—identified by International Classification of Diseases 9^th^ Revision Clinical Modification discharge diagnosis codes on inpatient claims. Operational definitions^52–56^, including quantitative measures of algorithm performance, are presented in Table 3.

**Table 3:** Operational definition of composite outcome of interest.

Outcome component	Diagnosis descriptor	ICD-9-CM diagnosis code(s)*	Diagnosis position and type/Claim type	PPV/Sensitivity
Venous thromboembolism	Iatrogenic pulmonary embolism and infarction	415.11	Principal position inpatient discharge diagnosis/MAX inpatient, short-stay MedPAR, or long-stay MedPAR claim	~95%/~77%^52^
	Other pulmonary embolism and infarction	415.19		
	Phlebitis and thrombophlebitis of femoral vein	451.11		
	Other phlebitis and thrombophlebitis	451.19		
	Phlebitis and thrombophlebitis of lower extremities, unspecified	451.2		
	Phlebitis and thrombophlebitis of unspecified site	451.9		
	Thrombophlebitis migrans	453.1		
	Embolism and thrombosis of inferior vena cava	453.2		
	Acute venous embolism and thrombosis of deep vessels of lower extremity	453.4X		
	Acute venous embolism and thrombosis of other specified veins	453.8X		
	Embolism and thrombosis of unspecified site	453.9		
Ischemic stroke	Occlusion and stenosis of precerebral arteries**	433.X1		~88%/~74%^56^
	Occlusion of cerebral arteries	434.X^†^		
	Acute, but ill-defined, cerebrovascular disease	436.X		

ICD-9-CM = International Classification of Diseases 9^th^ Revision Clinical Modification; MAX = Medicaid Analytic Extract; MedPAR = Medicare Provider Analysis and Review; PPV = positive predictive value.

*X indicates wildcard, i.e., values ranging from 0–9.

**Excluding mentions of cerebral infarction.

^†^Excluding 434.X0.

### Analytic approach and statistical analysis

For each self-controlled case series study, we constructed an analytic file in which the unit of observation was the person-day of observation time. The dependent variable was an indicator for outcome. Independent variables included a unique subject identifier, the subject’s observation period, the segment within the observation period, and other time-varying covariates discussed above. The primary analysis examined outcome incidence during the entire risk segment(s) vs. incidence during the non-risk segment(s). We used conditional Poisson regression models to estimate incidence rate ratios and 95% confidence intervals^33,48^. We conducted numerous secondary analyses (Appendix Table 5) to examine the robustness of our findings and assess potential violations of the design’s underlying assumptions as a measure of good practice^35^. We calculated ratios of incidence rate ratios in which the effect estimate for each antihyperlipidemic of interest was in the numerator and the effect estimate for pravastatin (as the prespecified referent) was in the denominator.

Analyses were conducted using SAS (SAS Institute Inc.: Cary, NC). The research described herein was approved via expedited mechanism by the institutional review board of the University of Pennsylvania. As the research was determined to be no greater than minimal risk, the board issued a waiver of informed consent. Methods related to human subjects research were developed and carried out in accordance with relevant guidelines and regulations.

### Data availability

Data that support study findings are available from the Centers for Medicare and Medicaid Services. Restrictions apply to the availability of these data, which were used by this study under a permissive data use agreement, and so are not publicly available. However, data may be available from the authors upon reasonable request and with permission from the Centers for Medicare and Medicaid Services.

## Electronic supplementary material

Appendix
