# Supplementary material for: Thromboembolic and neurologic sequelae of discontinuation of an antihyperlipidemic drug during ongoing warfarin therapy
Source: Sci Rep. 2017 Dec 21;7:18037. doi: 10.1038/s41598-017-18318-6 (PMC5740131; doi:10.1038/s41598-017-18318-6)
Supplement: Supplementary file 1 — Appendix [file 41598_2017_18318_MOESM1_ESM.pdf]

SUPPLEMENTARY INFORMATION

Supplementary information for "Thromboembolic and neurologic sequelae of discontinuation of an antihyperlipidemic drug during ongoing warfarin therapy" by Charles E. Leonard, Colleen M. Brensinger, Warren B. Bilker, Stephen E. Kimmel, Heather J. Whitaker, and Sean Hennessy

<u>Appendix Figure 1</u>. Depicted example of an offset drug-drug interaction (DDI) in which concomitancy was object drug triggered

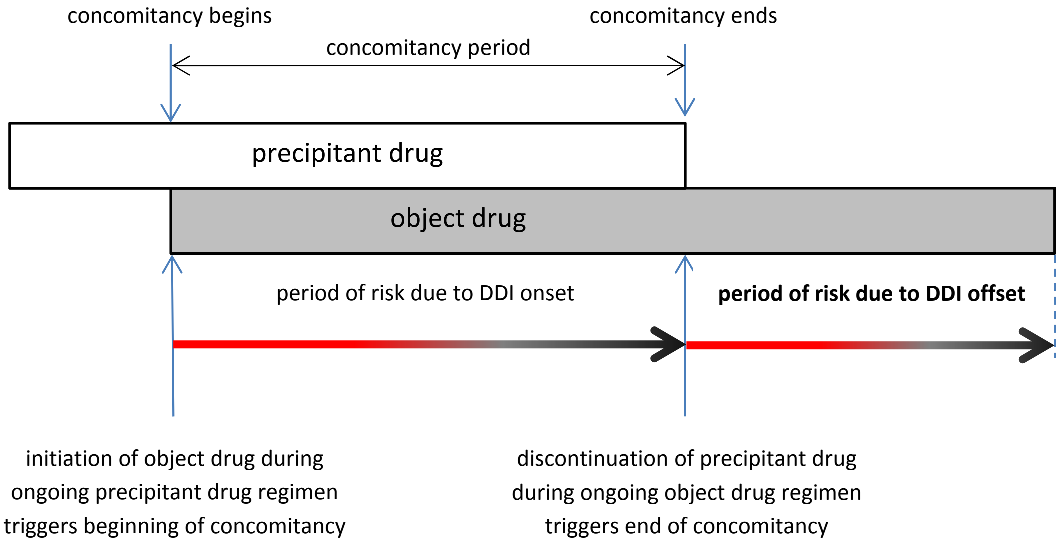

<u>Appendix Table 1.</u> Risk of venous thromboembolism/ischemic stroke within 90 days of discontinuing an antihyperlipidemic of interest in the presence of ongoing warfarin therapy

	Each antihyperlipidemic		Ratio of antihyperlipidemic of interest to pravastatin	
	Crude IRR	Adjusted IRR	Crude IRR	Adjusted IRR
atorvastatin	0.55 (0.34–0.89)	0.72 (0.43–1.20)	0.69 (0.26–1.82)	0.53 (0.18–1.53)
fenofibrate	0.32 (0.06–1.68)	0.27 (0.05–1.48)	0.41 (0.06–2.58)	0.20 (0.03–1.38)
gemfibrozil	0.83 (0.06–11.2)	2.16 (0.06–75.0)	1.05 (0.07–16.1)	1.59 (0.04–62.2)
lovastatin	0.43 (0.11–1.62)	0.41 (0.10–1.69)	0.54 (0.11–2.59)	0.30 (0.06–1.64)
pravastatin	0.79 (0.34–1.83)	1.36 (0.54–3.45)	referent	referent
rosuvastatin	0.28 (0.03–2.80)	0.21 (0.02–2.82)	0.36 (0.03–4.09)	0.16 (0.01–2.44)
simvastatin	0.68 (0.41–1.12)	0.91 (0.52–1.59)	0.85 (0.32–2.26)	0.67 (0.23–1.99)
IRR = incidence rate ratio				

Appendix Table 2. Findings from prespecified and post hoc secondary analyses, in which pravastatin findings were not set as the referent

Analysis*		Adjusted IRRs (95% CIs) for antihyperlipidemic of interest						
		fibrates		statins				
		feno	gem	rosuva	atorva	prava	lova	simva
Further elucidating the association between antihyperlipidemic discontinuation and outcome								
Stratify risk segment	Days 1–30	0.43 (0.08-2.43)	8.93 (0.19-414.8)	0.37 (0.04-3.84)	0.76 (0.44-1.33)	1.75 (0.68-4.46)	0.51 (0.13-1.91)	0.74 (0.39-1.41)
	Days 31–60	ND	ND	ND	0.51 (0.19-1.41)	ND	ND	2.21 (0.94-5.22)
	Days 61–90	ND	ND	ND	0.62 (0.20-1.98)	1.52 (0.14-16.0)	ND	0.33 (0.04-2.92)
Deconstruct composite outcome	VTE	0.15 (0.02-1.37)	1.44 (0.06-34.0)	0.35 (0.02-6.15)	0.43 (0.22-0.83)	0.99 (0.33-2.96)	0.62 (0.15-2.56)	0.65 (0.32-1.32)
	IS	1.43 (0.09-24.1)	ND	ND	2.67 (1.14-6.71)	3.16 (0.51-19.7)	ND	1.47 (0.62-3.52)
Lump antihyperlipidemics of interest by likelihood of CYP2C9 inhibition**		0.66 (0.41-1.05)				0.93 (0.59-1.46)		
Lump antihyperlipidemics of interest by likelihood of interacting with warfarin, per Truven Micromedex and Facts & Comparisons DDI module ratings**		0.70 (0.44-1.13)			0.82 (0.53-1.27)		†	
Assessing SCCS underlying assumptions, minimizing the role of bias and/or confounding								
Increase maximum length of risk segment from 90 to 120 days††		0.30 (0.05-1.68)	1.75 (0.03-94.1)	0.33 (0.04-2.71)	0.74 (0.45-1.20)	1.60 (0.64-4.00)	0.63 (0.18-2.14)	0.85 (0.49-1.50)
Exclude segments occurring before the first risk segment (i.e., conduct left-censored unidirectional SCCS)		ND	ND	ND	0.49 (0.17-1.40)	0.53 (0.06-5.08)	ND	0.44 (0.17-1.14)
Exclude segments occurring after the first risk and indeterminate risk segments (i.e., conduct right-censored unidirectional SCCS)		0.21 (0.03-1.57)	2.03 (0.06-72.8)	0.41 (0.03-5.25)	0.66 (0.38-1.15)	0.80 (0.29-2.18)	0.68 (0.13-3.48)	0.81 (0.44-1.49)
Reclassify second or later risk and indeterminate risk segments as non-risk segments		0.31 (0.05-1.79)	2.16 (0.06-75.0)	0.41 (0.03-5.34)	0.77 (0.46-1.30)	1.43 (0.56-3.66)	0.61 (0.16-2.26)	0.95 (0.54-1.67)
Include average daily dose of warfarin as covariate in outcome model		0.23 (0.03-1.48)	8.09 (0.32-205.8)	0.33 (0.02-4.42)	0.85 (0.49-1.49)	0.95 (0.32-2.77)	0.35 (0.08-1.52)	0.91 (0.50-1.67)
Exclude subjects with >1 outcome during the observation period		0.33 (0.06-1.82)	2.16 (0.06-75.0)	0.30 (0.02-3.77)	0.77 (0.43-1.36)	0.89 (0.31-2.59)	0.66 (0.16-2.75)	1.05 (0.59-1.90)
Exclude subjects that die during the observation period		0.31 (0.05-1.73)	1.72 (0.06-46.5)	0.21 (0.02-2.82)	0.62 (0.36-1.06)	1.45 (0.56-3.77)	0.49 (0.12-2.01)	0.91 (0.51-1.62)

atorva = atorvastatin; CI = confidence interval; CYP = cytochrome P450; DDI = drug-drug interaction; feno = fenofibrate; gem = gemfibrozil; IRR = incidence rate ratio; IS = ischemic stroke; lova = lovastatin; ND = not detectable / model produced unstable estimates; prava = pravastatin; rosuva = rosuvastatin; SCCS = self-controlled case series; simva = simvastatin; VTE = venous thromboembolism

*examining VTE/IS as composite outcome, unless otherwise noted

**post hoc analysis

†IRR for combined fenofibrate/gemfibrozil/rosuvastatin/lovastatin/simvastatin listed in merged fenofibrate-gemfibrozil-rosuvastatin cell

††thereby increases maximum length of indeterminate risk period from 90 to 120 days

Appendix Table 3. Summary of prior findings

Author and publication year / PubMed ID	Design	Setting / study period	Objective / outcome measure(s)	Findings
Zhelyazkova- Savova et al. 2014 / 24790817	Cross-sectional; "observational retrospective study"	Patients of cardiology clinic of a teaching University hospital in Varna, Bulgaria / 2007–2008	Investigate incidence of co-prescriptions involving statins and compare exposure of outpatients and inpatients to potential statin-drug interactions / Primary outcome measure: proportion of patients exposed to statin co-prescriptions with potentially-interacting drugs at hospital admission and discharge; secondary outcome measure: laboratory evidence supporting possible statin-drug interactions	Of 1,641 hospitalized patients, 572 were prescribed a statin either at hospital admission or discharge. Exposure to potential statin-drug interactions was similar at hospital admission (26.1%) and discharge (24.4%). Of all interacting drugs, acenocoumarol was most commonly found; the proportions of statin-acenocoumarol co-prescriptions were similar at hospital entry (11.5%) and discharge (12.4%). In seven patients of 69 exposed to the combination, INR was found to be higher in three, indicating a risk of over-anticoagulation. With another patient, the discontinuation of atorvastatin was followed by a decrease of INR from 3.1 to 2.4.
McDonald et al. 2012 / 22398967	Cross-sectional; "retrospective clinical evaluation / study"	Patients of a University-affiliated anticoagulation clinic / 2001–2007	Determine mean and range of warfarin dose change required to maintain an INR of 2–3 after amiodarone was added or discontinued in a cohort of patients on stable warfarin therapy / Outcome: mean change in warfarin dose	73 patients had an out of range INR attributed to a warfarin-amiodarone drug interaction. The mean change in warfarin dose required to maintain an INR of 2–3 was 25.6%. Warfarin dosing change requirements were highly variable, with a range of 5.9% to 65%. 27 of 73 patients had amiodarone discontinued. Mean warfarin dose prior to discontinuation of amiodarone was 3.9mg/day. Mean warfarin dose after discontinuation of amiodarone was 4.8mg/day.

Appendix Table 4. Time-varying covariates included in conditional Poisson regression models

Covariate category	Covariate component	Identification method
major non-chronic risk factors for VTE	VTE in prior 90 days	ICD-9-CM diagnosis codes
	hospital discharge on current day or in prior 90 days	MAX inpatient, MedPAR claims
major non-chronic risk factor for IS	IS in prior 90 days	ICD-9-CM diagnosis codes
drugs that may increase risk of VTE alone*	selective serotonin reuptake inhibitors / selective norepinephrine reuptake inhibitors on current day or in prior 30 days	NDC
drugs that may increase risk of IS alone*	testosterone on current day or in prior 30 days	NDC
	dexamethasone on current day or in prior 30 days	NDC
	methylprednisolone on current day or in prior 30 days	NDC
	epoetin alfa / darbepoetin alfa on current day or in prior 30 days	NDC
	filgrastim / sargramostim on current day or in prior 30 days	NDC
	diethylstilbestrol on current day or in prior 30 days	NDC, HCPCS
	cyproterone on current day or in prior 30 days	NDC
	flutamide on current day or in prior 30 days	NDC
	goserelin on current day or in prior 30 days	NDC, HCPCS
	leuprolide on current day or in prior 30 days	NDC, HCPCS
	raloxifene on current day or in prior 30 days	NDC
	toremifene on current day or in prior 30 days	NDC
	anastrozole on current day or in prior 30 days	NDC
	megestrol on current day or in prior 30 days	NDC
	cyclosporine on current day or in prior 30 days	NDC, HCPCS
	infliximab on current day or in prior 30 days	NDC, HCPCS
	immune globulins on current day or in prior 30 days	NDC, HCPCS
	interferon gamma-1b on current day or in prior 30 days	NDC, HCPCS
	interferon alfa-2a on current day or in prior 30 days	NDC, HCPCS
	muromonab on current day or in prior 30 days	NDC, HCPCS
	sirolimus / tacrolimus on current day or in prior 30 days	NDC, HCPCS
	aldesleukin on current day or in prior 30 days	NDC, HCPCS
	asparaginase on current day or in prior 30 days	NDC, HCPCS
	basiliximab on current day or in prior 30 days	NDC, HCPCS
	bevacizumab on current day or in prior 30 days	NDC, HCPCS
	bleomycin on current day or in prior 30 days	NDC, HCPCS
	carboplatin on current day or in prior 30 days	NDC, HCPCS
	denileukin on current day or in prior 30 days	NDC, HCPCS
	docetaxel on current day or in prior 30 days	NDC, HCPCS
	estramustine on current day or in prior 30 days	NDC
	fluorouracil on current day or in prior 30 days	NDC, HCPCS
	imatinib on current day or in prior 30 days	NDC
	irinotecan on current day or in prior 30 days	NDC, HCPCS

	lenalidomide on current day or in prior 30 days	NDC
	paclitaxel on current day or in prior 30 days	NDC, HCPCS
	thalidomide on current day or in prior 30 days	NDC
	heparins, including low molecular weight heparins on current day or in prior 30 days	NDC, HCPCS
	pentosan on current day or in prior 30 days	NDC
	chlorpromazine on current day or in prior 30 days	NDC, HCPCS
	clozapine on current day or in prior 30 days	NDC
	olanzapine on current day or in prior 30 days	NDC, HCPCS
	quetiapine on current day or in prior 30 days	NDC
	risperidone on current day or in prior 30 days	NDC, HCPCS
	thioridazine on current day or in prior 30 days	NDC
	celecoxib on current day or in prior 30 days	NDC
	acetohydroxamic acid on current day or in prior 14 days	NDC
	botulinum toxins on current day or in prior 30 days	NDC, HCPCS
	papaverine on current day or in prior 30 days	NDC, HCPCS
	topiramate on current day or in prior 30 days	NDC
	tretinoin on current day or in prior 30 days	NDC
drugs that may increase risk of VTE and IS*	oral contraceptive/hormone replacement therapy on current day or in prior 30 days	NDC
	nonsteroidal anti-inflammatory drug/cyclooxygenase-2 inhibitor on current day or in prior 30 days	NDC
	tamoxifen on current day or in prior 30 days	NDC
	nicotine replacement therapy on current day or in prior 30 days	NDC
	recombinant factor VIIa on current day or in prior 30 days	NDC, HCPCS
	cisplatin on current day or in prior 30 days	NDC, HCPCS
major non-chronic diseases that may affect coagulation	acute infection on current day or in prior 14 days	ICD-9-CM diagnosis codes
drugs that may affect coagulation	non-warfarin oral anticoagulant on current day or in prior 30 days	NDC
	subcutaneous/injectable anticoagulant identifiable in ambulatory claims on current day or in prior 30 days	NDC, HCPCS
	oral antiplatelet agent on current day or in prior 30 days	NDC
	aspirin on current day or in prior 30 days	NDC
drugs that may interact with warfarin	oral drugs that can interact with warfarin, per Truven Health Analytics Micromedex Solutions, on current day or in prior 30 days: amiodarone, mirtazapine, naproxen, capecitabine, procarbazine, imatinib, fluvoxamine, dronedarone, teriflunomide, cyclophosphamide, methotrexate, erlotinib, leflunomide, etoposide, vilazodone, paroxetine, lomitapide, rivaroxaban, torsemide, oxandrolone, mercaptopurine, valproic acid, entacapone, proguanil, ketoprofen, dapsone, aprepitant, enzalutamide, prasugrel, methyltestosterone, celecoxib, ropinirole	NDC
	oral drugs that can interact with warfarin, per Truven Health Analytics Micromedex Solutions , on current day or in prior 14 days: levofloxacin, moxifloxacin, erythromycin, cloxacillin, sulfamethoxazole, dicloxacillin, fluconazole, ampicillin, ceftibuten, telithromycin, sulfisoxazole, ciprofloxacin, ketoconazole, gemifloxacin, penicillin, posaconazole, itraconazole, oseltamivir, azithromycin, cefpodoxime, carbenicillin, cefdinir, clarithromycin, ofloxacin, amoxicillin, norfloxacin, cefixime, cephradine, enoxacin, cefadroxil, cephalexin, nalidixic acid, metronidazole, gatifloxacin	NDC
	oral “clinically relevant” CYP2C9 inhibitors, per Indiana University’s Clinical Pharmacology drug interaction	NDC

	website, on current day or in prior 30 days: amiodarone, efavirenz, isoniazid, paroxetine	
	oral “clinically relevant” CYP2C9 inhibitors, per Indiana University’s Clinical Pharmacology drug interaction website, on current day or in prior 14 days: fluconazole, metronidazole, sulfamethoxazole, voriconazole	NDC
	oral “clinically relevant” CYP2C9 inducers, per Indiana University’s Clinical Pharmacology drug interaction website, on current day or in prior 30 days: carbamazepine, nevirapine, phenobarbital, rifampin	NDC
therapeutic drug monitoring for warfarin	International Normalized Ratio testing/monitoring, prothrombin time testing/monitoring	CPT, HCPCS
average daily warfarin dose**	defined by (prescription quantity x warfarin tablet strength) / prescription days’ supply	NDC, days’ supply

CPT = current procedural terminology; CYP = cytochrome p450; HCPCS = healthcare common procedure coding system; ICD-9-CM = international classification of diseases 9th revision clinical modification; IS = ischemic stroke; MAX = Medicaid analytic extract; MedPAR = Medicare provider analysis and review; NDC = national drug code; VTE = venous thromboembolism
* per Tisdale & Miller’s Drug-induced diseases: prevention, detection, and management. Limited to drugs with evidence level A or B
** included in secondary analysis

Appendix Table 5. Prespecified and post hoc secondary analyses

Analysis	Rationale
Further elucidating the association between antihyperlipidemic discontinuation and outcome	
Stratify risk segment into days 1-30, 31-60, and 61-90	Facilitates elucidation of duration-response effects
Deconstruct composite outcome and examine VTE and IS separately	Facilitates elucidation of differential effects by outcome component
Lump antihyperlipidemics of interest by likelihood of CYP2C9 inhibition (i.e., [atorvastatin, fenofibrate, gemfibrozil, rosuvastatin] vs. [lovastatin, pravastatin, simvastatin])*	Facilitates elucidation of potential underlying mechanism; increases statistical precision
Lump antihyperlipidemics of interest by likelihood of interacting with warfarin, per Truven Micromedex and Facts & Comparisons DDI module ratings (i.e., [fenofibrate, gemfibrozil, lovastatin, rosuvastatin, simvastatin] vs. [atorvastatin, pravastatin])*	Facilitates elucidation of potential underlying mechanism; increases statistical precision
Assessing SCCS underlying assumptions, minimizing the role of bias and/or confounding	
Increase maximum length of risk segment from 90 to 120 days**	Examines robustness of findings to specification of risk segment length
Exclude segments occurring before the first risk segment (i.e., conduct left-censored unidirectional SCCS)	Minimizes reverse causality bias, although may be prone to exposure-trend bias
Exclude segments occurring after the first risk and indeterminate risk segments (i.e., conduct right-censored unidirectional SCCS)	Minimizes reverse causality bias, although may be prone to exposure-trend bias
Reclassify second or later risk and indeterminate risk segments as non-risk segments	Considers effect of depletion of susceptible risk segment
Include average daily dose of warfarin as covariate in outcome model	Controls for potential confounding by time-varying confounding by warfarin dose
Exclude subjects with >1 outcome during the observation period	Assesses impact of potential failure of outcome independence assumption; second or later outcomes, if influenced by initial outcome, may cause failure of underlying Poisson model
Exclude subjects that die during the observation period	Alleviates concern for death as an ultimate interferent event (and thereby censoring observation time), especially since the outcome of interest increases mortality; alleviates concern for immortal time bias
CYP = cytochrome P450; DDI = drug-drug interaction; IS = ischemic stroke; SCCS = self-controlled case series; VTE = venous thromboembolism<br>* post hoc analysis<br>** thereby increases maximum length of indeterminate risk period from 90 to 120 days	
